# Classification of Non-Small Cell Lung Cancer’s Tumor Immune Micro-Environment and Strategies to Augment Its Response to Immune Checkpoint Blockade

**DOI:** 10.3390/cancers13122924

**Published:** 2021-06-11

**Authors:** Alexander Chi, Xia He, Lin Hou, Nam P. Nguyen, Guangying Zhu, Robert B. Cameron, Jay M. Lee

**Affiliations:** 1 Department of Radiation Oncology, Beijing Chest Hospital, Capital Medical University, Beijing 101100, China; 2 Department of Radiation Oncology, Jiangsu Cancer Hospital, Nanjing Medical University, Nanjing 210009, China; 3 Center for Statistical Science, Tsinghua University, Beijing 100084, China; houl@tsinghua.edu.cn; 4 Department of Radiation Oncology, Howard University, Washington, DC 20060, USA; namphong.nguyen@yahoo.com; 5 Department of Radiation Oncology, China-Japan Friendship Hospital, Beijing 100029, China; zryyfa@163.com; 6 Division of Thoracic Surgery, Department of Surgery, University of California at Los Angeles, Los Angeles, CA 90095, USA; rcameron@mednet.ucla.edu (R.B.C.); JaymoonLee@mednet.ucla.edu (J.M.L.)

**Keywords:** NSCLC, LUAD, LUSC, tumor immune microenvironment, immune checkpoint blockade

## Abstract

**Simple Summary:**

Immune checkpoint blockade (ICB) has become a major treatment for lung cancer. Better understanding of the tumor immune micro-environment (TIME) in non-small cell lung cancer (NSCLC) is urgently needed to better treat it with this type of therapy. In this review, we describe and explore how NSCLC’s TIME relates to response to ICB, as well as how to treat those with unresponsive types of TIME, which will significantly impact future research in lung cancer immunotherapy.

**Abstract:**

Immune checkpoint blockade (ICB) with checkpoint inhibitors has led to significant and durable response in a subset of patients with advanced stage EGFR and ALK wild-type non-small cell lung cancer (NSCLC). This has been consistently shown to be correlated with the unique characteristics of each patient’s tumor immune micro-environment (TIME), including the composition and distribution of the tumor immune cell infiltrate; the expression of various checkpoints by tumor and immune cells, such as PD-L1; and the presence of various cytokines and chemokines. In this review, the classification of various types of TIME that are present in NSCLC and their correlation with response to ICB in NSCLC are discussed. This is conducted with a focus on the characteristics and identifiable biomarkers of different TIME subtypes that may also be used to predict NSCLC’s clinical response to ICB. Finally, treatment strategies to augment response to ICB in NSCLC with unresponsive types of TIME are explored.

## 1. Introduction

The systemic therapeutic options for advanced-stage non-small cell lung cancer (NSCLC) have expanded greatly in recent years to include not only chemotherapy and targeted therapies but also immune checkpoint inhibitors (ICI) [1]. Clinical outcome in patients with PD-L1 expressing treatment-naïve stage IV or previously-treated NSCLC has significantly improved with the emergence of anti-PD-1 and anti-PD-L1 ICIs [2,3,4,5]. In the first-line setting, significant survival advantage over standard chemotherapy with anti-PD-1/anti-PD-L1(anti-PD-(L)1) monotherapy has been consistently observed in EGFR and ALK wild-type stage IV patients with tumor cell PD-L1 expression ≥ 50% [4,5]. For those with PD-L1 expression < 50%, combining an anti-PD-1 antibody with standard chemotherapy has become the first-line treatment of choice on the basis of both superior progression-free survival (PFS) and overall survival (OS) observed over standard chemotherapy in randomized controlled phase 3 trials [6,7]. In previously-treated EGFR and ALK wild-type patients with any PD-L1 expression, a survival advantage over chemotherapy from anti-PD-(L)1 monotherapy was also consistently found [8,9,10]. This advantage over chemotherapy appears to be largest in patients with high PD-L1-expressing tumors (tumor cells: ≥50%, or tumor infiltrating immune cells: ≥10%). In addition, durable response significantly longer than that of chemotherapy was observed in responders to anti-PD-(L)1 antibodies [9]. Overall, the majority of current clinical evidence demonstrated that EGFR and ALK wild-type advanced-stage NSCLC patients with high PD-L1-expressing tumors benefited the most from anti-PD-(L)1 ICIs, despite quantitative variations between the currently available PD-L1 immunohistochemistry (IHC) assays [5,11]. However, PD-L1 expression level alone does not always predict for response to anti-PD-(L)1 ICIs [12]. Independent from PD-L1, a high tumor mutational burden (TMB), which correlates with tumor neoantigen load and effector T cell interferon (IFN)-γ gene signatures, was also shown to correlate with therapeutic benefit from ICIs [5,12,13,14,15,16]. PD-L1 expression level, TMB, or effector T cell IFN-γ gene signatures may each correlate with certain characteristics of a tumor immune micro-environment (TIME) that will be optimal for PD-(L)1 immune checkpoint blockade (ICB). However, none of them alone can be used to reliably select for all responders to anti-PD-(L)1 ICIs. More thorough understanding of NSCLC’s TIME is required in order to select NSCLC patients more reliably for ICIs. In this review, the classification of different types of TIME that may exist in NSCLC and their characteristics are discussed in the context of NSCLC’s response to ICB. Furthermore, strategies to augment ICI’s therapeutic efficacy in NSCLC patients who respond poorly are explored.

## 2. TIME Classification Applicable to NSCLC and Its Correlation with Response to ICB

One of the major immune-inhibitory mechanisms in the tumor micro-environment is the upregulation of PD-1 expression in tumor-infiltrating lymphocytes (TILs), leading to CD8^+^ T cell suppression and regulatory T (T_reg_) cell proliferation upon interaction with its ligands (PD1 ligands 1 and 2: PD-L1 and PD-L2, respectively), which are upregulated on tumor cells through constitutive oncogenic signaling, or an adaptive response to interferon signaling-triggered antitumor immunity [17]. Because of this underlying mechanism, antitumor activity of the TILs can be restored through PD-(L)1 immune checkpoint blockade, and this has led to durable response in a subset of patients with different solid tumors [18,19]. In NSCLC’s tumor microenvironment (TME), PD-L1 can be expressed in tumor and/or immune cells. Interestingly, response to anti-PD-L1 antibody has been correlated with PD-L1 expression in tumor-infiltrating immune cells, but not in tumor cells [19]. This is likely related to the removal of myeloid cell-mediated immune suppression, leading to increased T cell activation resulting from enhanced antigen presentation upon PD-(L)1 blockade [20,21,22,23]. The TIME of poor responders to anti-PD(L)1 therapy has initially been characterized into the following types on the basis of histological observations before and after treatment with an anti-PD-L1 antibody: little or no tumor-infiltrating immune cells (immunological ignorance), intra-tumoral immune cell infiltration with minimal or no PD-L1 expression (a non-functional immune response), and an excluded immune infiltrate around the outer edge of the tumor cell cluster [19]. These types of TIME have no evidence of functional effector T cells (Table 1).

Another TIME classification system that also applies to NSCLC has been proposed [24]. In this system, the TIME is classified by the level of tumor PD-L1 expression and TILs: type I, PD-L1+ and TILs+; type II, PD-L1- and TILs-; type III, PD-L1+ and TILs-; type IV, PD-L1- and TILs+ (Table 1). Type I TIME is consistent with a state of adaptive immune resistance with T cell exhaustion mediated by the PD-1–PD-L1 inhibitory immune axis, which has been effectively targeted with anti-PD-(L)-1 blockade. Here, PD-(L)1 expression in the tumor-infiltrating CD8^+^ T cells has been essential to PD-(L)1 ICI therapeutic efficacy [19,25,26]. Type II TIME, which represents a state of immunological ignorance, has been associated with a lack of response to ICB [19,24]. Type III TIME represents a state of constitutive PD-L1 expression on tumor cells resulting from oncogenic signaling pathway activation, which is more prevalent in oncogenic mutation-driven cancers, such as adenocarcinoma of the lung (LUAD). Increased PD-L1 expression has been observed on NSCLC cells with activating gene alterations in KRAS, EGFR, and ALK, which has been associated with upregulated MAPK, PI3K–AKT–mTOR signaling, and JAK–STAT3 activation [27,28,29,30,31,32,33]. However, such expression is not due to the presence of functional TILs [34]. Subsequently, response to anti-PD-(L)1 ICIs alone is poor, despite PD-L1 expression in tumor cells. This has been reported in NSCLC patients with EGFR mutations and ALK rearrangements, which are also associated with low tumor neoantigen load [35,36]. Type IV TIME describes a state of ineffective IFN-γ signaling that fails to induce any PD-L1 expression [37], or an environment of immune exhaustion through additional immune checkpoints. For NSCLC, alternative immune checkpoints, such as B7x and HHLA2, were found to be expressed in the majority of PD-L1-negative cases, which inhibited T cell receptor (TCR)-mediated CD4^+^, CD8^+^ T cell proliferation, and T cell cytokine production [38]. 

The four-type classification system captures the main features of a TIME responsive to PD-(L)1 immune check point blockade, a state of adaptive immune resistance or T cell exhaustion that relies heavily on the PD-(L)1 immune checkpoint: increased PD-(L)1 expression on tumor and immune cells, and prominent tumor infiltration by functional TILs. This type of TIME is also described as an “inflamed” TIME. On the other hand, the main feature of an unresponsive or “cold” TIME is a lack of functional TILs in the TIME, which can be characterized with a lack of TILs (type II: immunological ignorance, excluded infiltrate, or type III: intrinsic induction), or the presence of non-functional TILs (type IV: tolerance; non-functional immune response). These types of TIMEs are associated with or without PD-L1 expression, which further demonstrates the limitations of using PD-L1 expression alone to select patients for anti-PD-(L)1 ICIs and a need for treatment strategies to augment tumor response to ICIs in cancers with an unresponsive TIME. Overall, different TIME subtypes represent variations in different aspects or steps of antitumor immunity generation and maintenance, involving a variety of factors that are intrinsic to tumor cells and extrinsically present in the TME. They will all need to be further understood in order to better characterize the TIME and effectively target tumors with unresponsive types of TIME [39,40]. 

## 3. TIME Subtype Classification Based on Analysis of Immunogenomic Data from the Cancer Genome Atlas (TCGA) 

To further understand the cancer immune landscape, researchers used various immunogenomic methods to classify the TIME across 33 cancers into the wound-healing, IFN-γ-dominant, inflammatory, lymphocyte-depleted, immunologically quiet, and TGF-β-dominant subtypes on the basis of the distinct distribution of five immune-oncologic gene signatures (macrophages/monocytes, lymphocyte infiltrate, TGF-β response, IFN-γ response, and wound healing) [41]. Their characteristics are summarized in Table 2. 

The wound healing, IFN-γ-dominant, and inflammatory subtypes are associated with relatively higher lymphocyte fractions (LF), which is the highest in the IFN-γ-dominant TIME. Type II helper T cells (Th2) and regulatory T cells (Tregs) were also elevated in the wound healing and IFN-γ-dominant TIME subtypes, as observed in a TGF-β-dominant TIME. The lymphocyte-depleted, immunologically quiet, and TGF-β-dominant TIME subtypes are associated with noticeably higher fractions of M2 macrophages and lower fractions of M1 macrophages. The highest and lowest M1/M2 ratios were observed in the IFN-γ-dominant and the immunologically quiet subtypes, respectively. Overall, the inflammatory subtype was associated with the best overall survival (OS). Only increased LF in the wound healing and IFN-γ dominant TIMEs significantly correlated with increased OS. This was likely related to the lower tumor proliferation rate associated with the inflammatory TIME. The lymphocyte-depleted, immunologically quiet, and TGF-β-dominant subtypes were associated with lower LF, worse survival, and higher incidence of progression. Factors associated with increased immune activation, such as lymphocyte infiltration, TCR richness, and increased fractions of Th17 and Th1 cells are associated with improved survival, while features of immune suppression, such as the wound healing (high angiogenic gene expression), macrophage regulation, and TGF-β signatures are associated with shortened survival [41]. 

The proportions of different TIME subtypes vary substantially among different cancers. The inflammatory, IFN-γ-dominant, and wound-healing subtypes are most common in lung adenocarcinoma (LUAD), while wound-healing and IFN-γ-dominant subtypes predominate in lung squamous cell carcinoma (LUSC). The immunologically quiet TIME is absent in both LUAD and LUSC. Consistent with their predominant TIME subtypes, LUAD and LUSC have the highest leukocyte fractions among all solid tumors analyzed, which partially explains their response to ICIs [9,41,42,43]. Increases in lymphocyte and macrophage signatures are associated with increased OS for LUAD and prolonged progression-free interval (PFI) for both LUAD and LUSC. This is most likely related to the increased fractions of CD8^+^ T cells and M1 macrophages in their predominant TIME subtypes. When broken down to specific immune cells, monocytes, mast cells (resting), dendritic cells (DCs), and memory B cells are prominently associated with prolonged OS for LUAD, whereas Tfh cells, γδ T cells, CD8^+^ T cells, activated NK cells, and M1 macrophages are associated with prolonged OS for LUSC. Tregs, CD8^+^ T cells, CD4 T cells, resting mast cells, M1 macrophages, DCs (resting), and memory B cells are associated with prolonged PFI for both LUAD and LUSC, thus suggesting the importance of an overall active immune infiltrate for achieving a durable response and prolonged survival after ICB in lung cancer patients. 

The tumor neo-antigen load is highest in the wound healing and IFN-γ dominant TIMEs and lowest in the immunologically quiet TIME. Higher tumor neo-antigen loads in the first two types of TIMEs are associated with increased PFI, but the opposite has been observed in the inflammatory, lymphocyte-depleted, and immunologically quiet TIME subtypes [41]. This finding may relate to the presence of a normal adaptive antitumor immune response to increased tumor neo-antigens in the first two TIME subtypes but the presence of immune tolerance and immunological ignorance/exclusion in the latter three TIME subtypes. The way in which the level of tumor neoantigens associates with the level of TILs in each TIME subtype remains to be further investigated. Among all factors of immunogenicity, elevated SNV neoantigen load, non-silent mutations, and intra-tumoral heterogeneity (ITH) generally correlate with increased leukocyte fraction within the TIME. This usually represents elevated CD8^+^ T cells, M1 macrophages, and CD4^+^ memory T cells, and decreased Treg, mast, DC, and memory B cells. These correlations are strongest for in an inflammatory TIME, with weaker correlations observed in the wound healing, IFN-γ dominant, and the lymphocyte depleted TIMEs. 

Different levels of driver mutation enrichment are found in different TIME subtypes, with most of them identified in the wound healing and IFN-γ dominant TIMEs, which are also predominant TIME subtypes in LUSC and LUAD. These alterations are associated with different levels of tumor neoantigens and/or the expression of various immunomodulators (IMs) (Table 3). 

Some are associated with increased leukocyte fraction (TP53, HLA-B, BRAF, PTEN, NF1, APC, and CASP8), while others are associated with decreased leukocyte fraction (IDH1 R132H, GATA3, KRAS, NRAS, CTNNB1, and NOTCH1). Their association with tumor neoantigen generation, IM expression, and ultimately leukocyte fraction provides further evidence for tumor intrinsic gene alterations’ role in the sculpting of the TIME, which warrants further exploration to guide the treatment of NSCLC and other solid tumors [41]. 

The pattern of IM expression varies in different TIME subtypes. Stimulatory modulator CXCL10 is most highly expressed in the IFN-γ-dominant TIME, while inhibitory modulators, such as EDNRB and BTLA, are most highly expressed in the more immune-suppressive TIME subtypes. A balance between T cell activation and suppression is found in more immune-stimulatory TIME subtypes, which is evidenced by the expression of both stimulatory and inhibitory IM genes, such as SLAMF7, TNFSF4 (OX40L), IL10, CD40, and IDO1. On the contrary, modulators associated with immune infiltration are more frequently deleted in the immunologically quiet TIME (e.g., TGFB1, KIR2DL1, KIR2DL3), which is consistent with a lack of TILs in this TIME subtype. Overall, TIME subtypes with increased CD8^+^ T cell infiltration have been associated with the expression of stimulatory IMs, while those with increased infiltration by CD4 T cells and macrophages were associated with increased TGF-β signaling (Table 2). This pattern of IM expression reflects the predominance of different extracellular signaling networks associated with the fraction of different immune cells in the TIME [41]. 

Intrinsic tumor mutations interact with external signaling networks in a particular TIME with different driver mutations modulating IM expression in a TIME subtype-specific manner through common transcription factors (TFs). For example, ATM mutations and co-occurring STK11 and SMARCA4 mutations may drive wound healing TIME-specific gene expression through STAT5A in LUAD, while KEAP1 mutations, which often co-occur with STK11 and SMARCA4 mutations, drive the expression of genes specific to the immunologically quiet and TGF-β-dominant TIMEs through IRF8 in LUAD [41,44]. In LUSC, NFE2L2 mutation may drive the expression of wound healing and IFN-γ-dominant TIME-specific genes through IRF4, as well as the TGF-β dominant TIME specific gene expression through NFKB2 [41]. TIME characterization may be further enhanced with identifying T cell associated receptors and ligands that are uniquely present or absent in particular TIME subtypes, such as the absence of CTLA, LAG-3, TIM-3, TIGIT, ICOS, and IL2A in the inflammatory TIME, or the presence of IL1B and VEGFB in the TGF-β dominant TIME [41]. 

## 4. Potential Biomarkers and Therapeutic Targets in ICI Responsive vs. Nonresponsive TIME with a Focus on NSCLC

The presence of functional TILs is critical to antitumor immunity, and response to ICI largely rests on the presence of a TIME with increased TILs, as well as increased expression of PD-(L)1 in both tumor and immune cells, especially early during treatment [19,45,46,47,48]. Additional features include increased CD4 T cells, M1/M2 ratio, IFN-γ signaling, tumor neoantigen load/TMB, and upregulation of various stimulatory and inhibitory IMs [19,47,48,49,50,51,52,53]. These features are most consistent with that of an IFN-γ dominant TIME, while many of them are also present in the inflammatory and the wound healing TIMEs (Table 2). These TIME subtypes represent the predominant TIME subtypes in NSCLC. They are also enriched with many driver mutations, many of which are associated with increased leukocyte fraction within the TIME (Table 3). All these features may serve as biomarkers to identify TIME subtypes that are associated with response to ICI in NSCLC. 

As a result of tumor-driven tolerance, poor antitumor immunity and a lack of response to ICI may be observed, even in the presence of tumor-associated antigen (TAA)-specific T cells [54]. This may be due to tumor-induced T cell apoptosis, T cell suppression by suppressive cytokines, such as TGFβ, or suppressive immune cells, such as Tregs or myeloid-derived suppressor cells (MDSCs), as well as altered expression of stimulatory or inhibitory immune checkpoints or modulators on T cells and other immune cells [54,55,56,57,58,59,60,61]. In NSCLC, tolerance commonly occurs with low or no PD-L1 expression on tumor cells due to impaired IFN signaling, or the activation of alternative immune checkpoints [62,63]. The impaired IFN-γ signaling can be associated with inactivating JAK mutations, which may be enriched in a wound healing TIME subtype (Table 3). These mutations result in impaired MHC I upregulation, tumor cell proliferation, and poor response to anti-PD-(L)1 blockade, partly attributing to a paucity of tumor cell PD-L1 expression [37,64]. Therefore, inactivating JAK mutations may serve as a biomarker for poor response to ICI in the presence of TILs. This may be especially useful in LUSC or LUAD, which often present with a wound-healing TIME. They are also targetable therapeutically, as JAK-independent mechanisms of pro-inflammatory pathway activation can be induced. For instance, activation of the STING pathway can be induced by STING agonists, chemotherapy, or radiotherapy in the presence of intra-tumoral dendritic cells [65,66,67,68]. Dysfunctional TILs in the absence of tumor PD-L1 expression may also represent increased sub-population of terminally exhausted CD8^+^ T cells, which are immune-suppressed through multiple immune checkpoints other than the PD-(L)1 checkpoint [38,69,70,71,72,73,74,75,76,77]. These terminally exhausted CD8^+^ cells may be identified by their expression of multiple immune checkpoints and modulators other than PD-(L)1 [73,74,75,76,77,78,79,80]. In TIME’s possession of many characteristics of immune tolerance (Table 2 and Table 3), such as the TGF-β-dominant and wound healing TIMEs, combining inhibitors to these immune checkpoints and modulators may be a viable treatment strategy, which has been shown to be feasible pre-clinically with the simultaneous blockade of PD-1 and TIM-3 [81].

A paucity of TILs with or without PD-L1 expression in the TME, a state of “immunological ignorance” or “excluded infiltrate”, has been associated with a poor response to ICIs. Such a state is most consistent with an immunologically quiet TIME, which is often associated with slowly proliferating tumors, or a lymphocyte depleted TIME, which is more often observed in fast proliferating tumors, such as NSCLC [82]. These TIME subtypes are mainly characterized by the lowest levels of any immune infiltrate with the lowest fraction of TILs but the highest fractions of macrophages (highest M2/M1 ratio). In addition, they are associated with relatively lower level of tumor neoantigen load, TCR diversity, CXCL10, and the highest level of EDNRB (Table 2), which are suggestive of an overall immunosuppressive TME with poor immunogenicity and T cell trafficking. IDH1 mutation, a mutation commonly associated with a non-inflamed immune phenotype, is most commonly found in the immunologically quiet and the lymphocyte depleted TIMEs [83]. It accounts for the majority of mutation-associated neoantigens (MANAs) in these TIME subtypes. EGFR mutation is enriched in the lymphocyte-depleted TIME, which has been associated with low baseline PD-L1 expression and low levels of functional cytotoxic T lymphocytes, despite a state of constitutive PD-L1 expression in the presence of activating EGFR mutations [27,34,84]. Other mutations associated with poor T cell infiltration, such as mutations in the Wnt/β-catenin pathway, have also been identified in these TIME subtypes [85,86,87,88]. These mutations, along with TIME composition, may help identify immunologically ignorant/excluded TIME subtypes. However, the immunologically quiet TIME largely pertains to slow-growing gliomas and was not identified in the TCGA NSCLC samples [41].

Immunological ignorance/exclusion may result from a lack of tumor neoantigens, as well as impairments in antigen presentation, along with T cell priming/activation, tumor trafficking, or infiltration [40,89]. These mechanisms of immune suppression are commonly identified in NSCLC. Despite a relatively high tumor-associated antigen (TAA) load associated with NSCLC at baseline, its TAA profile may change overtime and after treatment as a result of immune editing due to the loss of tumor subclones or chromosomal loss of truncal mutations, leading to the elimination of immunity-generating clonal TAAs [90,91,92,93]. Immune editing in NSCLC may also be associated with the presence of intrinsic tumor mutations. For instance, the lack of an adaptive immune response in EGFR mutant LUAD has been associated with low levels of TAAs and T cell clonality, leading to poor T cell priming and activation [94]. Wnt/β-catenin activation due to mutations, somatic copy number alterations (SCNAs), or over-expression has also been identified in NSCLC, leading to immunological ignorant/exclusion caused by impaired antigen presentation, T cell priming/activation, and trafficking [85,86,87,88,95,96]. As shown in melanoma, β-catenin activation within tumor cells results in transcription repressor ATF3-induced suppression of CCL4 expression, causing poor DC recruitment and cross-presentation in the lymph nodes, as well as a lack of CXCL9/CXCL10-secreting DCs at the tumor site, leading to poor CD8^+^ effector T cell recruitment [86,95]. Moreover, β-catenin activation in DCs inhibits T cell priming by upregulation of mTOR-dependent IL-10 production [87]. However, β-catenin in DCs is required for T cell maintenance after clonal expansion.

Other causes of an immunologically ignorant/exclusive TIME, including deficits in tumor antigen presentation/tumor cell recognition through the loss of MHC I expression and/or the downregulation or inactivation of beta-2 microglobulin (B2M), a component of the MHC class I complex in the TME; impaired T cell extravasation and tumor infiltration through various routes of chemokine modulation; and tumor intrinsic factors, such as galectin-1, are also identified in NSCLC [97,98,99,100,101,102,103,104,105,106,107]. These impairments are largely caused by gene alterations in tumor intrinsic signaling pathways, some of which lead to a TIME with constitutive tumor PD-L1 expression but impaired T cell infiltration [108]. They include methylation of IFN-β-related genes, EGFR mutations, ALK re-arrangements, mutations or other types of gene alterations in the WNT/β-catenin pathway, PTEN loss, STK11/LKB1 mutations, KEAP1/NFE2L2 mutations, Myc over-expression, and rarely IDH1 mutations [27,28,29,30,31,32,33,34,35,44,85,86,94,96,101,108,109,110,111,112,113,114,115]. On the contrary, SMARCA4, TP53, and KRAS mutations have been associated with increased TILs and improved response to anti-PD-(L)1 in LUAD [116,117,118,119,120]. Their identification can not only enhance the characterization of each patient’s TIME but also aid in developing therapeutic strategies that may augment ICI efficacy in NSCLC [121,122].

Various cytokines have been shown to have an active role in T cell exclusion and immune suppression [123]. Among them, VEGF and TGF-β are commonly known to play a role in lung cancer progression [124,125]. VEGF induces T cell apoptosis through the stimulation of FasL expression in endothelial cells; inhibits T cell recruitment and adhesion through inhibiting TNFα-mediated expression of ICAM1, VCAM1, CXCL10, CXCL11; and stimulates the expression of suppressive immune checkpoints, such as PD-L1, CTLA4, TIM-3, and LAG-3 [102,126,127,128]. In addition, it is associated with the inhibition of DC maturation and increased Tregs and MDSCs in the TIME [129]. However, VEGF-induced T cell exclusion can be reversed by antiangiogenic agents, which leads to vessel normalization and increased T cell infiltration [127,130]. Increased angiogenesis does not correlate with the magnitude of tumor infiltration by T cells. As shown in the TCGA data, elevated angiogenesis may occur in both the wound healing and the lymphocyte-depleted TIME subtypes (Table 2), thus reflecting the co-existence of multiple mechanisms of immune suppression by VEGF and other angiogenic factors. This also makes VEGF and angiogenesis an important therapeutic target to enhance ICI’s efficacy, which has already been shown to be effective in NSCLC [131]. Another cytokine that also plays a critical role in immune suppression through multiple mechanisms, the TGF-β, is associated with a state of excluded immune infiltrate in the tumor stroma upon treatment with anti-PD(L)1 ICI, attributed to increased TGF-β signaling in peritumoral stromal cells [132,133,134]. Integrin αvβ8 expression on tumor cells may also lead to poor T cell infiltration through the activation of the TGF-β signaling pathway within suppressive immune cells, such as macrophages [135]. TGB-β-induced suppression of CD8^+^ T cells by other immune cells may be more commonly observed in the inflammatory and TGF-β-dominant TIME subtypes, which are associated with strong TGF-β signaling in various immune cells, such as CD4^+^ T cells, B cells, macrophages, and neutrophils, as well as relative abundance of M2 macrophages and Tregs. Increased TGF-β expression by CD8^+^ T cells in an IFN-γ-dominant TIME may also occur, possibly as a mechanism to balance increased IFN signaling [41]. Overall, elevated VEGF and TGF-β expression may potentially serve as biomarkers of not only T cell exclusion but also an overall state of immune suppression with increased infiltration of suppressive immune cells (Table 2) [129,136]. In addition, they may serve as potential therapeutic targets to augment response to ICI in NSCLC and other malignancies [128,130,131,132,133,134,137]. 

## 5. Therapeutic Strategies to Augment Response to ICI in NSCLC with “Unresponsive” Types of TIMEs 

**Figure 1:**
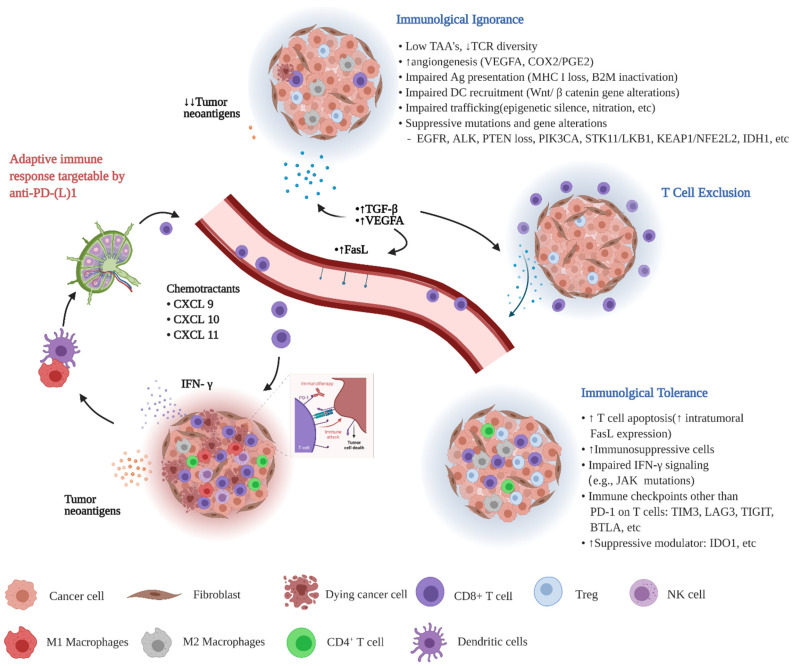
Characteristics of different types of TIME related to NSCLC’s response to ICI.

Overall, response to anti-PD-(L)1 ICI is dependent on the presence of a state of adaptive immune resistance resulting primarily from the activation of the PD-(L)1 immune checkpoint. This often manifests as a “hot or inflamed” TIME with high PD-L1 expression and effector CD8^+^ T cell infiltration. As in many other cancers, a lack of response in NSCLC is observed when the TIME is in a state of immune tolerance, ignorance, or T cell exclusion, which manifests as a lack of functional T cells or a paucity of any T cells in the TIME with or without PD-L1 expression on tumor cells (Figure 1). 

These cold TIME subtypes usually result from defects in different steps of antitumor-immunity generation resulting from tumor-intrinsic mutations, extrinsic factors such as suppressive cytokines and/or immune cells, or their interactions, depending on the specific tumor type and TIME subtype [40,44]. Identifying tumor features, such as the TIME subtype, particular mutations or gene alterations, and other biological features will most effectively predict patients’ response to ICI. Furthermore, it will guide the development of the most effective treatment strategy, which may maximize the efficacy of ICIs in cancer patients. This approach is especially helpful in NSCLC as its TIME is composed primarily of wound-healing, IFN-γ-dominant, or inflammatory subtypes. They are associated with high leukocyte infiltration, frequent genomic alterations/mutations, and other factors related to defects in antitumor-immunity generation and immune evasion [40,41,121,122]. Some of these characteristics can be used to both identify a cold tumor phenotype and be targeted therapeutically to augment the efficacy of ICIs [138,139,140,141]. 

In NSCLC, immune tolerance commonly results from loss-of-function JAK mutations; T cell exhaustion/apoptosis; increased expression of suppressive modulators and cytokines, such as IDO1 and TGF-β; and the suppression of normal CD8^+^ T cells’ interaction with tumor cells by Tregs and MDSCs [55,56,57,58,59,60,61,63,64,71,72,73,74,75,76,77,78,79,80,81]. These impairments reflect a state of T cell suppression/exhaustion and poor tumor cell recognition. They may be present in TIME subtypes with various magnitudes of CD8^+^ T cell infiltration or enriched with immunosuppressive cytokines, such as the IFN-γ-dominant or the TGF-β-dominant subtypes. When identified with a specific TIME subtype, treatment strategies combining ICI with other therapeutic agents, treatment modalities, or using other treatment approaches for immune activation may be developed to maximize antitumor response. For instance, activating the cGAS-STING pathway with radiotherapy, chemotherapy, STING agonists, or double-stranded (ds) DNA sensing-targeted nano-therapy along with adoptive T cell therapy may be considered in the presence of loss of function JAK mutations [64,65,66,67,68,141,142,143]. Among them, adoptive T cell therapy may also be combined with ICIs to provide a therapeutic solution to FasL-induced T cell apoptosis [144]. Cancer progression has been associated with increased terminal T cell exhaustion through additional immune checkpoints, such as LAG-3, TIM-3, TIGIT, and BTLA [73,74,75,76,77]. Inhibitors of these immune checkpoints have been shown to have antitumor activity and may improve treatment response in NSCLC with an overall unresponsive TIME if combined with anti-PD-(L)1 antibodies, other ICIs, or other treatment modalities [81,145,146,147,148,149,150,151]. Such a strategy can also be adopted when increased presence of suppressive modulators or immune cells is encountered [152,153,154,155,156,157,158,159,160]. Therefore, a combination strategy can effectively attack multiple immune defects simultaneously, which warrants further clinical investigations in NSCLC with tolerant TIME subtypes (Table 4). 

NSCLC with an immunologically ignorant/exclusion TIME has mainly been associated with immune editing, driver mutations, and gene alterations intrinsic to the tumor, as well as the presence of immune suppressants in the TIME. This reflects a strong influence of tumor-intrinsic factors on the TIME, as well as interactions between tumor-intrinsic factors and extrinsic signaling networks within the TME. Therefore, combining ICI with therapeutics targeting these factors may also be effective in this setting (Table 4). Among them, a lack of neoantigens due to immune editing can be partially overcome by cytotoxic therapy, such as chemotherapy or radiotherapy [138,141,161,162]. These treatments induce increased TAA generation and IFN-β production by tumor cells and DCs through STING pathway activation, leading to increased MHC I expression, DC recruitment, maturation, and antigen cross-presentation [141,163,164,165]. Radiotherapy has also been shown to broaden the TCR repertoire, enhance T cell trafficking, and normalize tumor vasculature at low doses [162]. These properties have made combining chemotherapy and/or radiotherapy with ICI a major treatment strategy for NSCLC, which has led to significant improvement in clinical outcome over traditional standard of care in the advanced setting [6,7,166,167,168,169]. Poor T cell recruitment due to increased VEGF-related angiogenic signaling may potentially be reversed by combining ICIs with VEGF inhibitors, COX inhibitors, and/or FasL antibodies, which will reduce T cell apoptosis, enhance T cell trafficking, and induce high endothelial venule formation [102,126,127,128,129,130]. Similarly, TGF-β inhibitors may reverse T cell exclusion due to cancer-associated fibroblast (CAF)-mediated TGF-β signaling in addition to reducing suppressive immune cells and their activity [134,152,170]. This makes them good therapeutic options to combine with ICI and other treatment modalities in NSCLC or other malignancies whenever increased TGF-β signaling is detected in the TME [170,171]. 

Somatic mutations are frequent in NSCLC [172]. Many of them play a significant role in sculpting the TIME of NSCLC [14,34,35,44,85,86,94,96,101,108,109,110,111,112,113,114,115,173]. Among them, alterations in EGFR, PIK3CA, STK11, and KEAP1 are most commonly found in LUAD, while PTEN loss and PIK3CA driver mutations have been more commonly observed in LUSC [122]. Also identified in LUAD, ALK re-arrangement has been associated with significantly poorer response to ICI when compared with other commonly known driver mutations [174]. RET re-arrangements have also been associated with poor response to ICI, which is likely due to the low TMB and PD-L1 expression observed in RET mutant LUAD [175]. However, this does not preclude RET-re-arranged patients from responding to ICIs [176]. In LUAD, STK11/LKB1 and KEAP1 mutations frequently co-occur with KRAS mutations and with each other, while CTNNB1 and PIK3CA mutations are associated with EGFR mutations [177]. STK11/LKB1 mutations in KRAS mutant LUAD have been associated with poor response to anti-PD-(L)1 ICI and other cytotoxic treatments, which may be associated with STK11/LKB1 and KEAP1 co-mutations’ association with STING suppression and metabolic reprogramming with enhanced glutamine dependence in KRAS mutant LUAD [44,111,112,178,179,180,181,182,183]. Glutaminase inhibition and KEAP1 expression have been shown to enhance radiosensitivity of KRAS mutant LUAD with STK11/LKB1 and KEAP1 mutations [184,185]. Whether these potential therapeutic options can potentiate sensitivity to ICI and the synergistic effect of combing ICI with cytotoxic therapies such as radiotherapy in KRAS mutant LUAD with STK11/LKB1 and/or KEAP1/NRF2 mutations warrants further exploration [166,184,185]. Other treatment options, such as an NRF2 inhibitor, can also be added into the treatment regimen for this very aggressive subgroup of LUAD that responds poorly to any treatments [186]. A similar approach targeting the Wnt/β catenin or the PI3K–AKT–mTOR pathways along with the PD-(L)1 pathway may be taken in the presence of CTNNB1 or PIK3CA mutations [29,187,188]. However, the majority of PIK3CA mutations are associated with other mutations [189]. Among them, EGFR mutations are known to be associated with poor immunogenicity, T cell tumor infiltration, and a lack of response to ICIs [34,94,174,190,191,192]. Similarly, ALK-re-arranged LUAD responds poorly to ICI, with only increased Treg in the TIME upon the development of TKI resistance or the treatment of TKI followed by ICI [35,174,193]. 

Different EGFR mutations may influence the TIME in LUAD differently, leading to variations in clinical response to ICI. For instance, EGFR exon 19 deletions have been associated with lower TMB than exon 21 L858R point mutation, and this has correlated with poorer response to ICI [192]. Moreover, T790M positivity has been associated with less PD-L1 expression and shorter PFS after ICI monotherapy in LUAD [174,194]. Exon 21 mutation appears to correlate with better response to ICI, thus making combining ICI and an EGFR TKI a potential treatment strategy in EGFR mutant LUAD [195]. However, ICI administration preceding or concurrent with osimertinib has been associated with significant pulmonary toxicity [196,197]. Similarly, the clinical feasibility of combining an ALK inhibitor with an ICI is largely limited by severe hepatic toxicities [198]. On the contrary, combining ICI, cytotoxic treatment modalities, and inhibitors of other targets in the TIME, such as VEGF, may be clinically feasible and effective in NSCLC [199]. This may be partially attributed to the increased generation of TAAs with cytotoxic agents and treatment modalities, such as chemotherapy and radiotherapy [162,169]. For mutations associated with low TMB, such as alterations in EGFR, ALK, and RET, this treatment strategy could be further explored in future clinical trials.

## 6. Conclusions

Different TIME subtypes exist in NSCLC. The presence of functional TILs is the most critical feature of a TIME associated with antitumor response to immune checkpoint blockade. Poor response to immune checkpoint blockade is associated with a TIME with identifiable features of immune tolerance, immunological ignorance, or T cell exclusion, which can all be identified in NSCLC. These features represent defects in the generation of antitumor immunity resulting from tumor intrinsic gene alterations, TIME-specific external signaling networks, or an interaction of the two. They may be used to not only identify NSCLC with unresponsive TIME subtypes, but also to guide the development of the more effective treatments that may augment ICI’s efficacy in NSCLC with such unresponsive phenotypes. Given cytotoxic treatment, such as radiotherapy’s immunostimulatory properties that may help overcome immunological tolerance and ignorance, the way in which to best combine it with ICI should be further explored.

## Figures and Tables

**Table 1:** General classification of the tumor immune micro-environment.

References	Method	Criteria	TIME Classification	Major Features	Additional Features
Herbst et al. [19]	IHC	PD-L1 expression	**Responsive**	**Before Rx**	**Before Rx**
		(TC and IC)		Increased PD-L1 expression	Increased expression of another checkpoint (NSCLC):
		CD8^+^ T cell infiltration		(TC, IC)	B7-H3, CTLA-4, TIM3, LAG3, IDO1, PD-L2
					Decreased CX3CL1; increased CTLA-4
					Increased IFN-γ and IFN-γ-inducible genes (e.g., IDO1 and CXCL9)
				**After Rx**	**After Rx**
				Increased PD-L1 expression	Increased tumor IFN-γ expression
				(TC, IC)	Gene expression pattern of immune activation:
				CD8 and Th1 T cell activation	granzyme-A, B; Perforin, EOMES, IFN-γ, TNF
					CXCL10, CD8A, CTLA 4
			**Non-Responsive**	**Pre-Rx and After Rx**	**After Rx**
			Immunological ignorance	Little or no TILs	No overexpression of genes associated with immune activation
			Non-functional immune response	TIL without PD-L1 expression	No overexpression of genes associated with immune activation
					(with pre-treatment CD 8 T cell infiltrate)
			Excluded infiltrate	Immune infiltrate at tumor margin	Same as the two types above, except with increased CTLA-4 expression
					Proliferation and PD-L1 expression in immune cells at tumor margin
Teng et al. [24]	IHC	PD-L1 expression (TC)	Type I (adaptive immune resistance)	PD-L1 (+), TIL (+)	Immunogenic mutations associated with
		TILs			increased TILs of higher PD-1, CTLA-4 expression
			Type II (immune ignorance)	PD-L1 (-), TIL (-)	No pre-existing T cell infiltration
			Type III (intrinsic induction)	PD-L1 (+), TIL (-)	More common in oncogenic mutation-driven NSCLC
					LUAD: PD-L1 expression-associated EGFR mutations
			Type IV (tolerance)	PD-L1 (-), TIL (+)	Increased myeloid cells
					Activation of other immune checkpoints and suppressive pathways

Rx: treatment.

**Table 2:** Characteristics the TCGA TIME subtype classification.

TIME Subtypes	Wound Healing ^ǂ^	IFN-γ Dominant	Inflammatory	Lymphocyte Depleted	Immunologically Quiet	TGF-β Dominant
Leukocyte fraction *	Intermed.	High	Intermed.	Low	Low	Highest
**Lymphocyte fraction (25–55%)**	High	Highest	High	Intermed. low	Lowest	Intermed.
**TIL (H and E)**	High	Highest	Intermed. low	Low	Lowest	Intermed.
**Immune cell composition**						
**T cells**						
CD8 T cells (<15%)	Intermed. high	Highest	High	Intermed. low	Lowest	Intermed.
CD4 T cells (<35%)						
Th1	Lowest		Elevated		Elevated	Elevated
Th2	Highest	Highest	Lowest	Intermed.	Low	Intermed. high
Tfh (<10%)	High	Highest	Intermed.	Low	Lowest	Intermed. low
Tregs (<5%)	High	Highest	Intermed. high	Low	Lowest	High
**Macrophages (38–60%)**				Elevated	Most elevated	Elevated
M0 (<15%)	Highest	High	Intermed. low	Intermed.	Lowest	High
M1 (<10%)	Intermed.	Highest	Intermed.	Intermed. low	Lowest	Intermed.
M2 (>20%)	Intermed. low	Lowest	Intermed.	High	Highest	High
**Tumor proliferation rate**	Highest	Highest	Low	High	Lowest	High
**Survival**						
OS	Intermediate	Intermediate	Best	Worst	Worse	Worst
PFI	Intermediate	Intermediate	Best	Worst	Worse	Worst
**NSCLC subtype**	Predom. in LUSC; third common in LUAD **	Second most common in LUAD and LUSC	Predom. In LUAD ***	LUSC **		
**Factors of immunogenecity**						
**DNA damage**						
Tumor neoantigen load						
SNVs	Highest	Second highest			Lowest	
Indels	Highest	Second highest			Lowest	
ITH	Elevated	Elevated	Lowest			
Enriched oncogenic driver mutations	APC, JAK1, PIK3CA, FGFR3	PIK3CA, FGFR3	CDH1, PIK3CA, FGFR3	EGFR		
TCR diversity	Intermediate	Highest	Intermediate	Low	Lowest	Highest
**Immunomodulators**						
Expression						
CXCL10		Highest			Lowest	Second Highest
EDNRB	Low	Lowest			Highest	
BTLA				High	High	
**Networks modulating the immune response**						
Predominant immune cells		CD8 T cells	CD8 T cells, CD4 T cells	CD4 T cells		CD4 T cells
Intracellular regulatory networks						
TGF-β (somatic mut+)		↓Leuk Fract.	↑Leuk Fract.			↓Leuk Fract.
	↑^r^ DC, M0, M1, M2, ^r^ NK, plasma cells	↑E, ^a^ Mast, M0/2, ^a^ DC, ^r^ NK, TγΔ	↑M1, M2, N, CD4, Treg	↑M0,M1, ^a^ DC	↑M0, Treg, ^mr^ CD4	↑^r^ DC
	↓^a^ NK, Treg, Tfh, CD8	↓CD8, Treg, Tfh, ^a^ NK	↓DC, M0, Tfh, ^m^ B cells, plamsa cells	↓monocytes	↓^n^ CD4, CD8	
Extracellular comm. networks						
		IFN-γ (+)	IFN-γ (+)			
		TGF-β (+)	TGF-β, TGF-βR(+)			TGF-β, TGF-βR(+)
T cell and macrophage-related signaling	CD80-CTLA4	LAG-3, CD27/28	CD27, PD-1	TLR4, VEGFB	TLR4	TLR4
	CD70-CD27	TIGIT, ICOS, CTLA, PD-1	CCR4, 5; CXCR3 DARC		EDN3-EDNRB, CX3CL1-CX3CR1	ITGB2
	IL1A/1B-IL1R2	CXCR3, CCR1,4,5				CD276
	CXCL9-CXCR3	BTLA				

^ǂ^ associated with high expression of angiogenic genes; ^a^: activated; ^r^: resting; ^m^: mature; ^n^: naïve; * highest in LUSC and LUAD (median: ≈30%); ** decreased survival; *** increased survival; ↑: increased; ↓: decreased.

**Table 3:** Mutations associated with the most common neoantigens and enriched in different TIME subtypes based on TCGA data.

TIME Subtype	Neoantigen-Related Driver Mutations	Enrichment
Wound healing	KRAS, KRAS G12, PIKC3A, TP53	**APC (OM)**, JAK1 (OM), TP53 *, FAT1, PPP2R1A, BRCA1, RB1, PIK3CA (OM), PTPRD, SPTA1, CTNNB1 *, FGFR3 * (OM), SMARCA4, KRAS G12, DACH1, PTEN *, SMARCA1, JAK1, KRAS *, MSH3
IFN-γ-dominant	PIKC3A, TP53	**CASP8**, HLA-A, **HLA-B**, ZNF750, TP53 *, MLH1, NF1 *, **FAT1**, PPP2R1A, BRCA1, RB1 *, PIK3CA(OM), PTPRD, **SPTA1**, DACH1
Inflammatory	BRAF	**BRAF**, CDH1 (OM), PBRM1 *
Lymphocyte-depleted	IDH1	**EGFR** (OM), *CTNNB1* *
Immunologically quiet	TP53, IDH1	*IDH1 R132H*, ATRX, CIC *, TP53 *
TGF-β-dominant	KRAS G12	KRAS G12

Bolded: associated with increased leuk. fraction; italics: associated with decreased leuk. fraction; *: associated with expression of known immunomodulators.

**Table 4:** Potential treatment strategies to enhance un-inflamed NSCLC’s response to ICI.

TIME Classification	Characteristics/Alterations	Proposed Treatment
**Tolerance**	JAK mutations	ICI + RT, chemotherapy, STING agonists, dsDNA sensing nano-therapy, and/or adoptive T cell transfer
	↑Tregs	ICI + Treg suppressors (e.g., anti-CD25, anti-ST2) ± RT
	↑MDSCs	ICI + MDSC suppressors (targeted therapies, HDAC inhibitors, CXCR1/2 inhibitors, etc.)
	↑FasL (MDSC)	ICI + adoptive T cell therapy or antibodies to FasL
	↑VEGF	ICI + VEGF inhibitors
	↑TGF-β	ICI + TGF-β inhibitors, fused anti-PD-L1/TGF-β trap
	↑IDO1	ICI + IDO1 inhibitors
	Terminal T cell exhaustion through other immune checkpoints	ICI of multiple/or alternative immune checkpoints, such as LAG-3, TIM-3, TIGIT, and BTLA ± RT ± chemotherapy
**Immunological Ignorance/Exclusion**		
	Lack of TAAs/immuno-editing	ICI + RT and/or chemotherapy
	STK11/LKB1, KEAP1 mutations	ICI + glutaminase inhibitors and/or NRF2 inhibitors ± RT, chemotherapy, or other STING activators
	Wnt/β-catenin mutations	Wnt/β-catenin inhibitors
	PTEN loss/PIK3CA mutations	PIK3CA inhibitors
	EGFR mutation (exon 21)	EGFR TKI with ICI at progression; ICI/chemo/inhibitors of other targets/RT combinations
	EGFR mutation (other)	ICI/chemo/inhibitors of other targets/RT combinations
	ALK or RET re-arranged	ICI/chemo/inhibitors of other targets/RT combinations
	↑Angiogenesis	ICI + VEGF inhibitors, COX-2 inhibitors, or FasL antibodies ± chemotherapy
	↑TGF-β	As above

TIME: tumor immune micro-environment; ↑: increased (in quantity, expression, or signaling).
